# 1-(4-Cyano­benz­yl)-3,5-dimethyl­pyridinium bis­(benzene-1,2-dithiol­ato)nickelate(III)

**DOI:** 10.1107/S1600536811004971

**Published:** 2011-02-16

**Authors:** Yan-Jie Dong, Xue-Jun Kong

**Affiliations:** a School of Chemistry and Chemical Engineering, Anqing Normal University, Anqing 246003, People’s Republic of China

## Abstract

The asymmetric unit of the title compound, (C_15_H_15_N_2_)[Ni(C_6_H_4_S_2_)_2_], contains half each of two independent centrosymmetric anions and a single cation in a general position. The Ni^III^ ions are coordinated by four S atoms in a square-planar geometry. The anions exhibit two packing modes, *viz*. stacked along the *a* axis in a face-to-face fashion with an alternate arrangement of anions and cations, and stacked in a side-by-side fashion, forming ribbons parallel to (011).

## Related literature

For general background to mol­ecular-based magnetic materials, see: Jones (1997 ▶); Akutagawa *et al.* (2009 ▶). For the role played by the size and shape of the counter-cations in determining the ground-state properties of the resulting materials, see: Ren *et al.* (2003 ▶). For related structures, see: Sellmann *et al.* (1991 ▶); Xie *et al.* (2002 ▶, 2003 ▶); Ren *et al.* (2002 ▶).
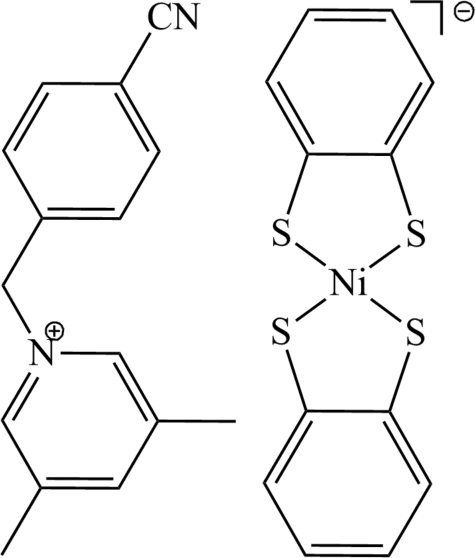


## Experimental

### 

#### Crystal data

(C_15_H_15_N_2_)[Ni(C_6_H_4_S_2_)_2_]
*M* *_r_* = 562.42
Triclinic, 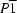

*a* = 7.1517 (7) Å
*b* = 12.8190 (13) Å
*c* = 15.3294 (16) Å
α = 69.774 (1)°
β = 77.740 (1)°
γ = 87.721 (1)°
*V* = 1287.8 (2) Å^3^
*Z* = 2
Mo *K*α radiation
μ = 1.10 mm^−1^
*T* = 296 K
0.36 × 0.30 × 0.28 mm

#### Data collection

Bruker SMART APEX CCD area-detector diffractometer
Absorption correction: multi-scan (*SADABS*; Bruker, 2000 ▶) *T*_min_ = 0.694, *T*_max_ = 0.749
6530 measured reflections
4530 independent reflections
3728 reflections with *I* > 2σ(*I*)
*R*_int_ = 0.042

#### Refinement

*R*[*F* ^2^ > 2σ(*F* ^2^)] = 0.036
*wR*(*F* ^2^) = 0.098
*S* = 1.02
4530 reflections
312 parameters
H-atom parameters constrained
Δρ_max_ = 0.25 e Å^−3^
Δρ_min_ = −0.39 e Å^−3^

### 

Data collection: *SMART* (Bruker, 2000 ▶); cell refinement: *SAINT* (Bruker, 2000 ▶); data reduction: *SAINT*; program(s) used to solve structure: *SHELXS97* (Sheldrick, 2008 ▶); program(s) used to refine structure: *SHELXL97* (Sheldrick, 2008 ▶); molecular graphics: *SHELXTL* (Sheldrick, 2008 ▶); software used to prepare material for publication: *SHELXTL*.

## Supplementary Material

Crystal structure: contains datablocks I, global. DOI: 10.1107/S1600536811004971/rz2554sup1.cif

Structure factors: contains datablocks I. DOI: 10.1107/S1600536811004971/rz2554Isup2.hkl

Additional supplementary materials:  crystallographic information; 3D view; checkCIF report

## Figures and Tables

**Fig. 1.**
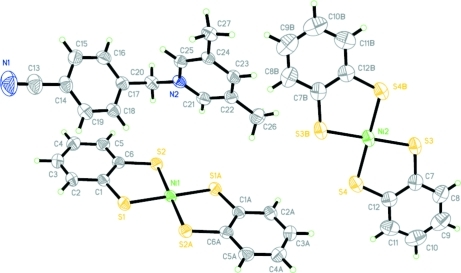
The molecular structure of (I) with the atom-numbering scheme. Displacement ellipsoids are drawn at the 30% probability level. Symmetry codes: (A) 1-x, 1-y, 1-z; (B) 1-x, 2-y, -z.

**Fig. 2.**
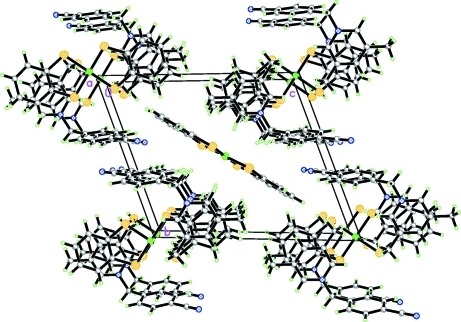
Packing diagram of (I) viewed along the a axis.

## supplementary crystallographic information

### Comment

Molecular solids with particular functionality are continuously paid much attention by chemists and physicists in the field of materials science (Jones, 1997). The preparation of new molecular based spin-bearing systems, among others, has been pursued from the viewpoint of materials/physical organic chemistry to develop novel molecular-based magnetic materials (Akutagawa *et al.*, 2009). In our previous research using benzylpyridinium derivatives ([RBzPy]^+^) as the counter-cation of [*M*(mnt)_2_]^-^ (where *M* = Ni, Pd and Pt and mnt^2-^ = maleodinitriledithiolate), a series of ion-pair compounds with segregated columnar stacks of cations and anions has been prepared (Ren *et al.*, 2002; Ren *et al.*, 2003; Xie *et al.*, 2002). The quasi one-dimensional magnetic nature of these compounds was attributed to intermolecular orbital interactions within the anionic columns. As an extension of our work on this series of complexes, we report here the crystal structure of the title compound, (I).

The asymmetric unit of (I) contains half each of two independent centrosymmetric [Ni(C_6_H_4_S_2_)_2_]^-^ anions and one (C_15_H_15_N_2_)^+^ cation. In the anions, the nickel(III) ions are coordinated by four S atoms in a square-planar geometry; the Ni—S bonds and S—Ni—S angles are in agreement with the corresponding values found in analogous complexes (Sellmann *et al.*, 1991; Xie *et al.*, 2003). The centrosymmetric [Ni(C_6_H_4_S_2_)_2_]^-^ anions are almost planar. The dihedral angle between the two benzene rings of the cation is 86.49 (6)°. In the crystal structure, the packing of the two anions is different (Fig. 2). The Ni1-containing anions stack in a side-by-side fashion, forming one-dimensional ribbons  parallel to (011); the shortest distance between the adjacent nickel(III) ions is 7.152 (6) Å. The Ni2-containing anions stack in a face-to-face fashion along the *a* axis with an alternating arrangement of [Ni(C_6_H_4_S_2_)_2_]^-^ anions and [C_15_H_15_N_2_]^+^ cations such that the pyridine ring of the cation lies above the benzene ring of the anion. The shortest distance between adjacent nickel(III) ions is also 7.152 (6) Å. A Ni···Ni distance of 8.155 (9)Å is found between adjacent Ni1-containing and Ni2-containing anions.

### Experimental

Benzene-1,2-dithiol (142 mg, 1.0 mmol) was added to a solution of sodium metal (46 mg, 2.0 mmol) in absolute ethanol (25 ml), under a nitrogen atmosphere at room temperature. A solution of NiCl_2_.6H_2_O (120 mg, 0.5 mmol) in ethanol (25 ml) was added, resulting in the mixture turning a muddy red-brown colour. Following this, [CNBzPy(CH_3_)_2_]Br (304 mg, 1.0 mmol) was added and the mixture allowed to stand with stirring for 1 h, and then stirred for an additional 24 h in air. The colour of the mixture gradually turned green, indicating oxidation from a dianionic species to the more stable monoanionic form. The precipitate was washed with absolute ethanol and diethyl ether and then dried. The crude product was recrystallized twice from dichloromethane to give the title compound (yield 198 mg, 54%).

### Refinement

All H atoms were placed in calculated positions and refined using a riding model, with C–H = 0.93–0.97 Å, and with *U*_iso_(H) = 1.2 *U*_eq_(C) or 1.5 *U*_eq_(C) for methyl H atoms.

### Crystal data

**Table:**

(C_15_H_15_N_2_)[Ni(C_6_H_4_S_2_)_2_]	*Z* = 2
*M**_r_* = 562.42	*F*(000) = 582
Triclinic, *P*1	*D*_x_ = 1.450 Mg m^−^^3^
Hall symbol: -P 1	Mo *K*α radiation, λ = 0.71073 Å
*a* = 7.1517 (7) Å	Cell parameters from 3577 reflections
*b* = 12.8190 (13) Å	θ = 2.6–27.0°
*c* = 15.3294 (16) Å	µ = 1.10 mm^−^^1^
α = 69.774 (1)°	*T* = 296 K
β = 77.740 (1)°	Block, dark green
γ = 87.721 (1)°	0.36 × 0.30 × 0.28 mm
*V* = 1287.8 (2) Å^3^	

### Data collection

**Table:**

Bruker SMART APEX CCD area-detector diffractometer	4530 independent reflections
Radiation source: sealed tube	3728 reflections with *I* > 2σ(*I*)
graphite	*R*_int_ = 0.042
φ and ω scans	θ_max_ = 25.1°, θ_min_ = 1.5°
Absorption correction: multi-scan (*SADABS*; Bruker, 2000)	*h* = −8→8
*T*_min_ = 0.694, *T*_max_ = 0.749	*k* = −12→15
6530 measured reflections	*l* = −18→15

### Refinement

**Table:**

Refinement on *F*^2^	Primary atom site location: structure-invariant direct methods
Least-squares matrix: full	Secondary atom site location: difference Fourier map
*R*[*F*^2^ > 2σ(*F*^2^)] = 0.036	Hydrogen site location: inferred from neighbouring sites
*wR*(*F*^2^) = 0.098	H-atom parameters constrained
*S* = 1.02	*w* = 1/[σ^2^(*F*_o_^2^) + (0.0581*P*)^2^] where *P* = (*F*_o_^2^ + 2*F*_c_^2^)/3
4530 reflections	(Δ/σ)_max_ = 0.001
312 parameters	Δρ_max_ = 0.25 e Å^−^^3^
0 restraints	Δρ_min_ = −0.39 e Å^−^^3^

### Special details

**Table:**

Geometry. All e.s.d.'s (except the e.s.d. in the dihedral angle between two l.s. planes) are estimated using the full covariance matrix. The cell e.s.d.'s are taken into account individually in the estimation of e.s.d.'s in distances, angles and torsion angles; correlations between e.s.d.'s in cell parameters are only used when they are defined by crystal symmetry. An approximate (isotropic) treatment of cell e.s.d.'s is used for estimating e.s.d.'s involving l.s. planes.
Refinement. Refinement of *F*^2^ against ALL reflections. The weighted *R*-factor *wR* and goodness of fit *S* are based on *F*^2^, conventional *R*-factors *R* are based on *F*, with *F* set to zero for negative *F*^2^. The threshold expression of *F*^2^ > σ(*F*^2^) is used only for calculating *R*-factors(gt) *etc*. and is not relevant to the choice of reflections for refinement. *R*-factors based on *F*^2^ are statistically about twice as large as those based on *F*, and *R*- factors based on ALL data will be even larger.

### Fractional atomic coordinates and isotropic or equivalent isotropic displacement parameters (Å^2^)

**Table:**

	*x*	*y*	*z*	*U*_iso_*/*U*_eq_	
Ni1	0.5000	0.5000	0.5000	0.05051 (14)	
Ni2	0.5000	1.0000	0.0000	0.06279 (16)	
S1	0.38833 (9)	0.43210 (6)	0.41084 (5)	0.06354 (19)	
S2	0.78410 (8)	0.44650 (5)	0.45826 (4)	0.05774 (17)	
S3	0.52163 (10)	0.85288 (5)	0.11833 (6)	0.0751 (2)	
S4	0.44027 (9)	1.09946 (6)	0.09034 (6)	0.0758 (2)	
C1	0.5817 (3)	0.3696 (2)	0.36074 (16)	0.0563 (6)	
C2	0.5622 (4)	0.3134 (2)	0.29922 (18)	0.0710 (7)	
H2	0.4429	0.3068	0.2862	0.085*	
C3	0.7171 (4)	0.2681 (3)	0.2582 (2)	0.0800 (8)	
H3	0.7024	0.2306	0.2177	0.096*	
C4	0.8974 (4)	0.2777 (2)	0.2765 (2)	0.0779 (8)	
H4	1.0026	0.2472	0.2480	0.093*	
C5	0.9189 (4)	0.3322 (2)	0.33656 (18)	0.0674 (7)	
H5	1.0394	0.3392	0.3482	0.081*	
C6	0.7616 (3)	0.37727 (19)	0.38061 (15)	0.0544 (5)	
C7	0.4721 (4)	0.8937 (2)	0.2174 (2)	0.0750 (8)	
C8	0.4671 (4)	0.8185 (3)	0.3089 (3)	0.0933 (10)	
H8	0.4884	0.7438	0.3178	0.112*	
C9	0.4315 (5)	0.8526 (4)	0.3851 (3)	0.1155 (14)	
H9	0.4298	0.8014	0.4456	0.139*	
C10	0.3978 (5)	0.9626 (5)	0.3733 (3)	0.1139 (14)	
H10	0.3753	0.9851	0.4261	0.137*	
C11	0.3969 (4)	1.0402 (3)	0.2847 (3)	0.0971 (10)	
H11	0.3712	1.1140	0.2779	0.116*	
C12	0.4356 (3)	1.0061 (2)	0.2045 (2)	0.0734 (7)	
C13	0.7251 (6)	0.5985 (3)	−0.0472 (2)	0.0964 (10)	
C14	0.8167 (5)	0.6096 (2)	0.0246 (2)	0.0708 (7)	
C15	1.0079 (5)	0.6423 (2)	0.0003 (2)	0.0809 (8)	
H15	1.0773	0.6544	−0.0612	0.097*	
C16	1.0958 (4)	0.6572 (2)	0.06717 (19)	0.0723 (7)	
H16	1.2245	0.6796	0.0505	0.087*	
C17	0.9938 (4)	0.63901 (18)	0.15919 (17)	0.0571 (6)	
C18	0.8028 (4)	0.60457 (19)	0.18304 (18)	0.0614 (6)	
H18	0.7338	0.5916	0.2447	0.074*	
C19	0.7138 (4)	0.5892 (2)	0.1172 (2)	0.0713 (7)	
H19	0.5858	0.5654	0.1343	0.086*	
C20	1.0872 (4)	0.65782 (19)	0.23205 (18)	0.0632 (6)	
H20A	1.0577	0.5944	0.2906	0.076*	
H20B	1.2250	0.6635	0.2093	0.076*	
C21	0.9853 (4)	0.7593 (2)	0.34242 (17)	0.0622 (6)	
H21	0.9952	0.6936	0.3918	0.075*	
C22	0.9338 (4)	0.8545 (2)	0.36168 (17)	0.0648 (7)	
C23	0.9208 (3)	0.9501 (2)	0.28705 (17)	0.0597 (6)	
H23	0.8853	1.0150	0.2995	0.072*	
C24	0.9592 (3)	0.95288 (18)	0.19364 (15)	0.0512 (5)	
C25	1.0102 (3)	0.85467 (18)	0.17933 (15)	0.0509 (5)	
H25	1.0374	0.8532	0.1177	0.061*	
C26	0.8910 (5)	0.8506 (3)	0.46412 (19)	0.0950 (10)	
H26A	0.8574	0.9231	0.4661	0.142*	
H26B	1.0023	0.8277	0.4907	0.142*	
H26C	0.7864	0.7985	0.5004	0.142*	
C27	0.9439 (3)	1.0558 (2)	0.11151 (17)	0.0617 (6)	
H27A	1.0669	1.0761	0.0692	0.093*	
H27B	0.9012	1.1153	0.1346	0.093*	
H27C	0.8536	1.0422	0.0781	0.093*	
N1	0.6563 (6)	0.5905 (3)	−0.1048 (2)	0.1353 (14)	
N2	1.0217 (3)	0.76078 (14)	0.25195 (12)	0.0528 (5)	

### Atomic displacement parameters (Å^2^)

**Table:**

	*U*^11^	*U*^22^	*U*^33^	*U*^12^	*U*^13^	*U*^23^
Ni1	0.0451 (2)	0.0580 (3)	0.0509 (2)	−0.00616 (18)	−0.01511 (17)	−0.01801 (19)
Ni2	0.0386 (2)	0.0489 (3)	0.0962 (4)	0.00137 (17)	−0.0198 (2)	−0.0160 (2)
S1	0.0478 (3)	0.0842 (4)	0.0722 (4)	−0.0036 (3)	−0.0202 (3)	−0.0387 (3)
S2	0.0473 (3)	0.0721 (4)	0.0593 (3)	−0.0029 (3)	−0.0192 (3)	−0.0244 (3)
S3	0.0622 (4)	0.0528 (4)	0.1011 (5)	0.0018 (3)	−0.0223 (4)	−0.0121 (3)
S4	0.0527 (4)	0.0594 (4)	0.1193 (6)	0.0036 (3)	−0.0267 (4)	−0.0308 (4)
C1	0.0559 (14)	0.0622 (14)	0.0501 (12)	−0.0090 (11)	−0.0119 (10)	−0.0171 (11)
C2	0.0641 (16)	0.0864 (18)	0.0730 (16)	−0.0116 (14)	−0.0170 (13)	−0.0376 (15)
C3	0.083 (2)	0.090 (2)	0.0773 (18)	−0.0109 (16)	−0.0060 (15)	−0.0465 (16)
C4	0.0702 (18)	0.088 (2)	0.0779 (18)	0.0033 (15)	−0.0050 (14)	−0.0381 (16)
C5	0.0548 (15)	0.0792 (18)	0.0669 (15)	0.0004 (12)	−0.0127 (12)	−0.0235 (14)
C6	0.0538 (13)	0.0553 (13)	0.0505 (12)	−0.0052 (10)	−0.0133 (10)	−0.0118 (10)
C7	0.0439 (14)	0.0775 (19)	0.095 (2)	−0.0151 (12)	−0.0167 (13)	−0.0157 (16)
C8	0.0681 (19)	0.099 (2)	0.095 (2)	−0.0250 (17)	−0.0201 (17)	−0.006 (2)
C9	0.082 (2)	0.150 (4)	0.098 (3)	−0.045 (3)	−0.019 (2)	−0.018 (3)
C10	0.077 (2)	0.165 (4)	0.102 (3)	−0.036 (3)	−0.007 (2)	−0.052 (3)
C11	0.0607 (18)	0.113 (3)	0.129 (3)	−0.0204 (17)	−0.0139 (19)	−0.057 (3)
C12	0.0415 (13)	0.0823 (19)	0.096 (2)	−0.0117 (12)	−0.0158 (13)	−0.0276 (16)
C13	0.131 (3)	0.077 (2)	0.090 (2)	0.0043 (19)	−0.047 (2)	−0.0267 (18)
C14	0.095 (2)	0.0486 (14)	0.0756 (17)	0.0042 (13)	−0.0351 (16)	−0.0201 (13)
C15	0.113 (3)	0.0626 (17)	0.0636 (16)	−0.0043 (16)	−0.0130 (16)	−0.0199 (13)
C16	0.0724 (18)	0.0616 (16)	0.0770 (18)	−0.0079 (13)	−0.0066 (14)	−0.0209 (14)
C17	0.0669 (15)	0.0398 (12)	0.0625 (14)	0.0040 (10)	−0.0202 (12)	−0.0113 (10)
C18	0.0655 (15)	0.0529 (14)	0.0629 (14)	0.0007 (11)	−0.0178 (12)	−0.0137 (11)
C19	0.0727 (17)	0.0596 (15)	0.0846 (19)	−0.0003 (13)	−0.0283 (15)	−0.0213 (14)
C20	0.0626 (15)	0.0515 (14)	0.0749 (16)	0.0057 (11)	−0.0262 (13)	−0.0145 (12)
C21	0.0633 (15)	0.0659 (16)	0.0537 (14)	−0.0124 (12)	−0.0227 (11)	−0.0084 (12)
C22	0.0615 (15)	0.0786 (18)	0.0563 (14)	−0.0147 (13)	−0.0149 (12)	−0.0226 (14)
C23	0.0522 (14)	0.0626 (15)	0.0682 (15)	−0.0056 (11)	−0.0131 (11)	−0.0265 (13)
C24	0.0393 (11)	0.0540 (13)	0.0609 (13)	−0.0056 (9)	−0.0159 (10)	−0.0168 (11)
C25	0.0479 (12)	0.0544 (13)	0.0508 (12)	−0.0033 (10)	−0.0187 (10)	−0.0132 (10)
C26	0.114 (3)	0.114 (3)	0.0588 (16)	−0.019 (2)	−0.0142 (16)	−0.0323 (17)
C27	0.0556 (14)	0.0527 (13)	0.0729 (15)	0.0009 (10)	−0.0198 (12)	−0.0130 (12)
N1	0.186 (4)	0.134 (3)	0.112 (2)	−0.004 (3)	−0.083 (3)	−0.044 (2)
N2	0.0505 (11)	0.0507 (11)	0.0562 (11)	−0.0044 (8)	−0.0207 (9)	−0.0109 (9)

### Geometric parameters (Å, °)

**Table:**

Ni1—S1	2.1459 (6)	C13—C14	1.443 (4)
Ni1—S1^i^	2.1459 (6)	C14—C15	1.380 (4)
Ni1—S2	2.1560 (6)	C14—C19	1.392 (4)
Ni1—S2^i^	2.1560 (6)	C15—C16	1.379 (4)
Ni2—S3^ii^	2.1451 (7)	C15—H15	0.9300
Ni2—S3	2.1451 (7)	C16—C17	1.388 (4)
Ni2—S4^ii^	2.1569 (8)	C16—H16	0.9300
Ni2—S4	2.1569 (8)	C17—C18	1.385 (3)
S1—C1	1.741 (3)	C17—C20	1.506 (3)
S2—C6	1.747 (2)	C18—C19	1.372 (3)
S3—C7	1.735 (3)	C18—H18	0.9300
S4—C12	1.739 (3)	C19—H19	0.9300
C1—C6	1.399 (3)	C20—N2	1.491 (3)
C1—C2	1.400 (3)	C20—H20A	0.9700
C2—C3	1.366 (4)	C20—H20B	0.9700
C2—H2	0.9300	C21—N2	1.349 (3)
C3—C4	1.395 (4)	C21—C22	1.370 (4)
C3—H3	0.9300	C21—H21	0.9300
C4—C5	1.368 (4)	C22—C23	1.373 (4)
C4—H4	0.9300	C22—C26	1.518 (4)
C5—C6	1.397 (4)	C23—C24	1.388 (3)
C5—H5	0.9300	C23—H23	0.9300
C7—C8	1.395 (4)	C24—C25	1.374 (3)
C7—C12	1.407 (4)	C24—C27	1.496 (3)
C8—C9	1.356 (5)	C25—N2	1.341 (3)
C8—H8	0.9300	C25—H25	0.9300
C9—C10	1.377 (6)	C26—H26A	0.9600
C9—H9	0.9300	C26—H26B	0.9600
C10—C11	1.379 (5)	C26—H26C	0.9600
C10—H10	0.9300	C27—H27A	0.9600
C11—C12	1.414 (4)	C27—H27B	0.9600
C11—H11	0.9300	C27—H27C	0.9600
C13—N1	1.133 (4)		
			
S1—Ni1—S1^i^	180.00 (3)	C15—C14—C13	119.2 (3)
S1—Ni1—S2	92.04 (2)	C19—C14—C13	120.8 (3)
S1^i^—Ni1—S2	87.97 (2)	C16—C15—C14	119.9 (3)
S1—Ni1—S2^i^	87.97 (2)	C16—C15—H15	120.0
S1^i^—Ni1—S2^i^	92.03 (2)	C14—C15—H15	120.0
S2—Ni1—S2^i^	180.0	C15—C16—C17	120.5 (3)
S3^ii^—Ni2—S3	180.00 (4)	C15—C16—H16	119.8
S3^ii^—Ni2—S4^ii^	91.75 (3)	C17—C16—H16	119.8
S3—Ni2—S4^ii^	88.25 (3)	C18—C17—C16	119.0 (2)
S3^ii^—Ni2—S4	88.25 (3)	C18—C17—C20	120.1 (2)
S3—Ni2—S4	91.75 (3)	C16—C17—C20	120.9 (2)
S4^ii^—Ni2—S4	180.00 (2)	C19—C18—C17	121.1 (2)
C1—S1—Ni1	104.89 (8)	C19—C18—H18	119.5
C6—S2—Ni1	104.81 (8)	C17—C18—H18	119.5
C7—S3—Ni2	105.49 (11)	C18—C19—C14	119.5 (3)
C12—S4—Ni2	104.82 (11)	C18—C19—H19	120.3
C6—C1—C2	119.0 (2)	C14—C19—H19	120.3
C6—C1—S1	119.51 (18)	N2—C20—C17	112.08 (18)
C2—C1—S1	121.47 (19)	N2—C20—H20A	109.2
C3—C2—C1	120.5 (3)	C17—C20—H20A	109.2
C3—C2—H2	119.7	N2—C20—H20B	109.2
C1—C2—H2	119.7	C17—C20—H20B	109.2
C2—C3—C4	120.5 (3)	H20A—C20—H20B	107.9
C2—C3—H3	119.8	N2—C21—C22	120.2 (2)
C4—C3—H3	119.8	N2—C21—H21	119.9
C5—C4—C3	119.7 (3)	C22—C21—H21	119.9
C5—C4—H4	120.1	C21—C22—C23	118.4 (2)
C3—C4—H4	120.1	C21—C22—C26	119.2 (3)
C4—C5—C6	120.7 (2)	C23—C22—C26	122.3 (3)
C4—C5—H5	119.7	C22—C23—C24	122.0 (2)
C6—C5—H5	119.7	C22—C23—H23	119.0
C5—C6—C1	119.5 (2)	C24—C23—H23	119.0
C5—C6—S2	121.75 (19)	C25—C24—C23	116.6 (2)
C1—C6—S2	118.69 (19)	C25—C24—C27	120.6 (2)
C8—C7—C12	119.3 (3)	C23—C24—C27	122.8 (2)
C8—C7—S3	122.0 (3)	N2—C25—C24	121.7 (2)
C12—C7—S3	118.7 (2)	N2—C25—H25	119.1
C9—C8—C7	121.0 (4)	C24—C25—H25	119.1
C9—C8—H8	119.5	C22—C26—H26A	109.5
C7—C8—H8	119.5	C22—C26—H26B	109.5
C8—C9—C10	120.2 (4)	H26A—C26—H26B	109.5
C8—C9—H9	119.9	C22—C26—H26C	109.5
C10—C9—H9	119.9	H26A—C26—H26C	109.5
C9—C10—C11	121.3 (4)	H26B—C26—H26C	109.5
C9—C10—H10	119.4	C24—C27—H27A	109.5
C11—C10—H10	119.4	C24—C27—H27B	109.5
C10—C11—C12	119.1 (4)	H27A—C27—H27B	109.5
C10—C11—H11	120.4	C24—C27—H27C	109.5
C12—C11—H11	120.4	H27A—C27—H27C	109.5
C7—C12—C11	119.0 (3)	H27B—C27—H27C	109.5
C7—C12—S4	119.2 (2)	C25—N2—C21	121.1 (2)
C11—C12—S4	121.8 (3)	C25—N2—C20	119.51 (19)
N1—C13—C14	178.7 (4)	C21—N2—C20	119.3 (2)
C15—C14—C19	120.0 (2)		
			
S2—Ni1—S1—C1	2.24 (8)	C8—C7—C12—S4	−179.55 (19)
S2^i^—Ni1—S1—C1	−177.76 (8)	S3—C7—C12—S4	0.3 (3)
S1—Ni1—S2—C6	−1.49 (8)	C10—C11—C12—C7	0.6 (4)
S1^i^—Ni1—S2—C6	178.51 (8)	C10—C11—C12—S4	−179.1 (2)
S4^ii^—Ni2—S3—C7	−177.93 (9)	Ni2—S4—C12—C7	1.4 (2)
S4—Ni2—S3—C7	2.07 (9)	Ni2—S4—C12—C11	−179.0 (2)
S3^ii^—Ni2—S4—C12	178.07 (8)	C19—C14—C15—C16	−1.4 (4)
S3—Ni2—S4—C12	−1.93 (8)	C13—C14—C15—C16	177.7 (3)
Ni1—S1—C1—C6	−2.8 (2)	C14—C15—C16—C17	0.3 (4)
Ni1—S1—C1—C2	178.49 (18)	C15—C16—C17—C18	0.7 (4)
C6—C1—C2—C3	−0.9 (4)	C15—C16—C17—C20	−178.4 (2)
S1—C1—C2—C3	177.8 (2)	C16—C17—C18—C19	−0.5 (4)
C1—C2—C3—C4	−0.4 (4)	C20—C17—C18—C19	178.6 (2)
C2—C3—C4—C5	0.5 (5)	C17—C18—C19—C14	−0.6 (4)
C3—C4—C5—C6	0.7 (4)	C15—C14—C19—C18	1.5 (4)
C4—C5—C6—C1	−2.0 (4)	C13—C14—C19—C18	−177.5 (3)
C4—C5—C6—S2	179.6 (2)	C18—C17—C20—N2	−72.8 (3)
C2—C1—C6—C5	2.1 (3)	C16—C17—C20—N2	106.3 (3)
S1—C1—C6—C5	−176.67 (18)	N2—C21—C22—C23	−0.1 (3)
C2—C1—C6—S2	−179.45 (18)	N2—C21—C22—C26	179.2 (2)
S1—C1—C6—S2	1.8 (3)	C21—C22—C23—C24	−0.3 (3)
Ni1—S2—C6—C5	178.62 (18)	C26—C22—C23—C24	−179.7 (2)
Ni1—S2—C6—C1	0.16 (19)	C22—C23—C24—C25	0.3 (3)
Ni2—S3—C7—C8	178.04 (19)	C22—C23—C24—C27	179.3 (2)
Ni2—S3—C7—C12	−1.8 (2)	C23—C24—C25—N2	0.2 (3)
C12—C7—C8—C9	−1.4 (4)	C27—C24—C25—N2	−178.85 (19)
S3—C7—C8—C9	178.8 (2)	C24—C25—N2—C21	−0.7 (3)
C7—C8—C9—C10	0.6 (5)	C24—C25—N2—C20	−177.0 (2)
C8—C9—C10—C11	0.8 (6)	C22—C21—N2—C25	0.6 (3)
C9—C10—C11—C12	−1.4 (5)	C22—C21—N2—C20	176.9 (2)
C8—C7—C12—C11	0.8 (4)	C17—C20—N2—C25	−44.8 (3)
S3—C7—C12—C11	−179.4 (2)	C17—C20—N2—C21	138.9 (2)

Symmetry codes: (i) −*x*+1, −*y*+1, −*z*+1; (ii) −*x*+1, −*y*+2, −*z*.
